# Adiponectin at Physiologically Relevant Concentrations Enhances the Vasorelaxative Effect of Acetylcholine via Cav-1/AdipoR-1 Signaling

**DOI:** 10.1371/journal.pone.0152247

**Published:** 2016-03-29

**Authors:** Yunhui Du, Rui Li, Wayne Bigond Lau, Jianli Zhao, Bernard Lopez, Theodore A. Christopher, Xin-Liang Ma, Yajing Wang

**Affiliations:** 1 Department of Physiology and Pathophysiology, Capital Medical University, Beijing 100069, China; 2 Department of Physiology, National Key Discipline and Key Laboratory of Cytophysiology of Shanxi Province, Shanxi Medical University, Shanxi, 030001, China; 3 Department of Emergency Medicine, Thomas Jefferson University, Philadelphia, Pennsylvania 19107, United States of America; Medical College of Wisconsin, UNITED STATES

## Abstract

Clinical studies have identified hypoadiponectinemia as an independent hypertension risk factor. It is known that adiponectin (APN) can directly cause vasodilation, but the doses required exceed physiologic levels several fold. In the current study, we determine the effect of physiologically relevant APN concentrations upon vascular tone, and investigate the mechanism(s) responsible. Physiologic APN concentrations alone induced no significant vasorelaxation. Interestingly, pretreatment of wild type mouse aortae with physiologic APN levels significantly enhanced acetylcholine (ACh)-induced vasorelaxation (P<0.01), an endothelium-dependent and nitric oxide (NO)-mediated process. Knockout of adiponectin receptor 1 (AdipoR1) or caveolin-1 (Cav-1, a cell signaling facilitating molecule), but not adiponectin receptor 2 (AdipoR2) abolished APN-enhanced ACh-induced vasorelaxation. Immunoblot assay revealed APN promoted the AdipoR1/Cav1 signaling complex in human endothelial cells. Treatment of HUVECs with physiologic APN concentrations caused significant eNOS phosphorylation and nitric oxide (NO) production (P<0.01), an effect abolished in knockdown of either AdipoR1 or Cav-1. Taken together, these data demonstrate for the first time physiologic APN levels enhance the vasorelaxative response to ACh by inducing NO production through AdipoR1/Cav-1 mediated signaling. In physiologic conditions, APN plays an important function of maintaining vascular tone.

## Introduction

Hypertension, a major risk factor of vascular dysfunction, is a significant cause of morbidity and mortality [1, 2]. Virtually every organ system of the body receiving circulatory flow is at risk for complications stemming from hypertension[3]. Despite extensive research in the past several decades, hypertension-related mortality continues to increase worldwide.

The vascular endothelium critically regulates circulatory function. Maintenance of endothelial homeostasis is essential for the prevention of vascular pathology, including hypertension [4]. Endothelial dysfunction refers to impairment of endothelium-dependent vasodilation, thereby disrupting endothelial homeostasis, leading to the pervasive clinical hypertensive phenotype. Complete comprehension of the underlying molecular mechanisms influencing impaired vascular relaxation, ultimately causing the endothelial dysfunction responsible for hypertension, may yield novel effective therapeutic targets.

It is well known that adiponectin (APN), an adipocyte-derived cytokine, protects the cardiovascular system against inflammation and improves endothelium-dependent vasodilation [5, 6]. Hypoadiponectinemia is an independent hypertension risk factor [7, 8]. Adiponectin knockout (APN^-/-^) mice manifest vascular endothelial dysfunction and increased blood pressure, reversed by APN administration [9, 10]. Previously, it has been demonstrated adiponectin can cause direct vasodilation [11, 12]. However, the adiponectin dose required to elicit such vasodilatory effects exceed physiologic concentrations at least by ten-fold. Heretofore, the role of physiologic APN levels in vascular hemostasis remains unclear.

We and others have previously demonstrated the vasculoprotective effects of adiponectin [9, 13]]. While supra-physiologic concentrations of APN have a direct vasodilatory effect, it is possible that physiologic APN concentrations may have a permissive “priming” effect upon endothelial cells to the vasodilatory effects of other agents. Therefore, the aims of the present study were 1) to determine the effect of physiologic APN concentrations upon vascular endothelial function, and 2) to investigate the molecular mechanisms responsible for any permissive vasodilatory effect observed of APN during concomitant administration with acetylcholine.

## Materials and Methods

### Mice

All experiments were performed on adult (8–10 week old) male AdipoR1 knockout mice (AdipoR1KO), AdipoR2 knockout mice (AdipoR2KO), Cav-1 knockout mice (Cav-1KO), or male wild type littermate controls (WT). The WT mouse strain was C57BL/6J, and AdipoR1KO mice were purchased from the Mutant Mouse Regional Resource Center (Chapel Hill, NC). All other mouse lines were purchased from Jackson Laboratory (Bar Harbor, Maine). Animals were housed at 22°C with a 12-hour light/12-hour dark cycle with free access to water and standard chow. Animals were euthanized under isoflurane anesthesia. All experiments were performed in adherence with the National Institutes of Health Guidelines on the use of Laboratory Animals, and were approved by the Thomas Jefferson University Committee on Animal Care.

### Aortic ring preparation and apparatus for assessment of vasorelaxation

Vessel chamber experiments were performed as previously described [9, 13]. Briefly, adult male WT, AdipoR1KO, AdipoR2KO, and Cav-1KO mice were anesthetized with 3% isoflurane. Descending aortic segments were isolated. Vascular segments were placed into ice-cold Krebs–Henseleit (K–H) buffer consisting of (mM): NaCl (118), KCl (4.75), CaCl_2_.2H20 (2.54), KH_2_PO_4_ (1.19), MgSO_4_.7H_2_O (1.19), NaHCO_3_ (25.0), and glucose (10.0). Aortic segments were carefully cleaned of fat and loose connective tissue. Segments were sectioned into rings (2–3 mm length), and mounted upon stainless steel hooks, aerated at 37°C in a 95% O_2_/5% CO_2_ mixture. Suspended in 1 ml of K-H tissue buffer, the aortic rings were mounted onto a Multi Mire Myograph System (620M, DMT-USA, Inc, Ann Arbor, MI) and vasculature constriction/dilation was recorder. The rings were stretched to an optimum preload of 0.4 g force (determined in preliminary experiments), and allowed to equilibrate for 60 minutes. During this period, the K–H buffer in the tissue bath was replaced every 15 minutes. Vascular ring tension was adjusted until 0.4 g preload force was maintained. After equilibration, the rings were exposed to 100 nM of U-46619 (9,11-epoxymethano-PGH2, Biomol Research Laboratories, Plymouth Meeting, PA) to ensure vascular smooth muscle stabilization (maximally effective concentration determined in preliminary experiments). After agonist washout, ring re-equilibration was permitted. Twenty minutes after the initial washing, 50nM of U-46619 was added to each ring bath to generate approximately 0.4 g vascular contraction. Once stable contraction was achieved, acetylcholine (ACh, a vasorelaxive agent eliciting endothelial NO production) was added to the bath in cumulative concentrations of 10^−8^–10^−4^ M to evaluate endothelial vasorelaxive function. After stabilization of ring response to cumulative ACh, bath washout and ring re-equilibration to baseline was permitted. The procedure was repeated with 10^−8^–10^−4^ M dose increments of acidified NaNO_2_ (prepared by dissolving NaNO_2_ in 0.1 N HCl titrated to pH 2.0), an agent eliciting vasorelaxation in an endothelium-independent manner. Any ring exhibiting less than 60% relaxation in response to maximal ACh concentrations was excluded from further study.

After achieving stable vasorelaxation by ACh, repeat solution bath washout and ring re-equilibration to baseline was permitted. Full-length APN (fAPN) was purchased from PeproTech Company (Rocky Hill, NJ). fAPN was added in cumulative concentrations of 5, 10, and 15μg/ml. To evaluate the involvement of NO in the observed vasorelaxive effects of fAPN, Nω-nitro-L-arginine methyl ester (L-NAME, a non-selective inhibitor of NO synthesis, dose 100 μmol/L), was administered concomitantly with fAPN.

### Cell culture and treatments

HUVECs (passage 2–3) were plated on six-well plates and cultured in endothelial growth medium containing 10% fatal bovine serum, 2mM glutamine, 100U/ml penicillin, and 100 μg/ml streptomycin at 37°C and 5% CO_2_. Upon reaching 80% confluence, cells were treated with vehicle or fAPN (5, 10, and 15 μg/ml). Cells were collected after 15, 30, or 60 minutes of fAPN treatment. Total nitric oxide (NO) content, phospho-eNOS, and total eNOS expression were determined as described in detail below.

### Small interfering RNA transfection

RNA oligonucleotides complementary to Cav1, AdipoR1, and AdipoR2 target sequences silenced respective gene expression. HUVECs were transfected with a siIMPORTER siRNA transfection kit (Qiagen Science Inc. Benelux, Venlo, Netherlands) per manufacturer’s protocol with siRNA duplexes against Cav1 (5’-UAUUAUGAGAUGGUAGGCAdTdT-3’), AdipoR1 (5’-GGACAACGACUAUCUGCUACATT-3’), AdipoR2 (5’-GGAGUUUCGUUUCAUGAUCGGTT-3’), and universal control oligonucleotides (AllStars, Westerville, OH). HUVECs were plated on six-well plates before transfection. After the HUVECs reached 80% confluence, siRNA was applied to each well (final concentration 50 nM).

### Immunoblotting and co-immunoprecipitation

HUVECs were lysed by cold lysis buffer and homogenized. After centrifugation, the supernatant was collected. For immunoblotting, proteins were separated on SDS-PAGE gels and transferred to PVDF membranes. Membranes were then incubated with primary antibodies and HRP-conjugated secondary antibody. The blot was developed with a Supersignal Chemiluminescence detection kit (Pierce, Rockford, IL). Bands were visualized by a Kodak Image Station 4000R Pro (Rochester, NY). For co-immunoprecipitation, cell lysates were pre-cleared with corresponding nonimmune IgG, and incubated together with protein A plus-Sepharose for 2 hours at 4°C. Cleaned lysates were then incubated with 2μg of anti-Cav1 antibody. Cell lysates were then incubated with protein A plus-Sepharose overnight at 4°C. Protein A beads were then extensively washed with lysis buffer. Proteins were eluted from beads, and resolved by elusion buffer. Samples with 2XSDS sample buffer were heated and separated by electrophoresis. After transfer to PVDF membranes, proteins were immunoblotted with anti-AdipoR1 as described above.

### Determination of total nitric oxide content in HUVECs

After treatment, HUVECs were collected and homogenized. Nitric oxide (NO) and its metabolic products (NO_2_ and NO_3_) in the supernatant (collectively known as NOx) were determined by a chemiluminescence NO detector (Siever 280i NO Analyzer), as described previously [14].

### Statistical analysis

All values in the text are presented as means±SEM of n independent experiments. All data (except Western blot density) were subjected to ANOVA followed by Tukey correction for post-hoc t test. Western blot densities were analyzed by the Kruskal-Wallis test, followed by Dunn’s post-hoc test. Probabilities of 0.05 or less were considered statistically significant.

## Results

### Physiologic APN doses facilitate ACh-induced vasorelaxation in an endothelium-dependent and NO-mediated manner

**Fig 1:**
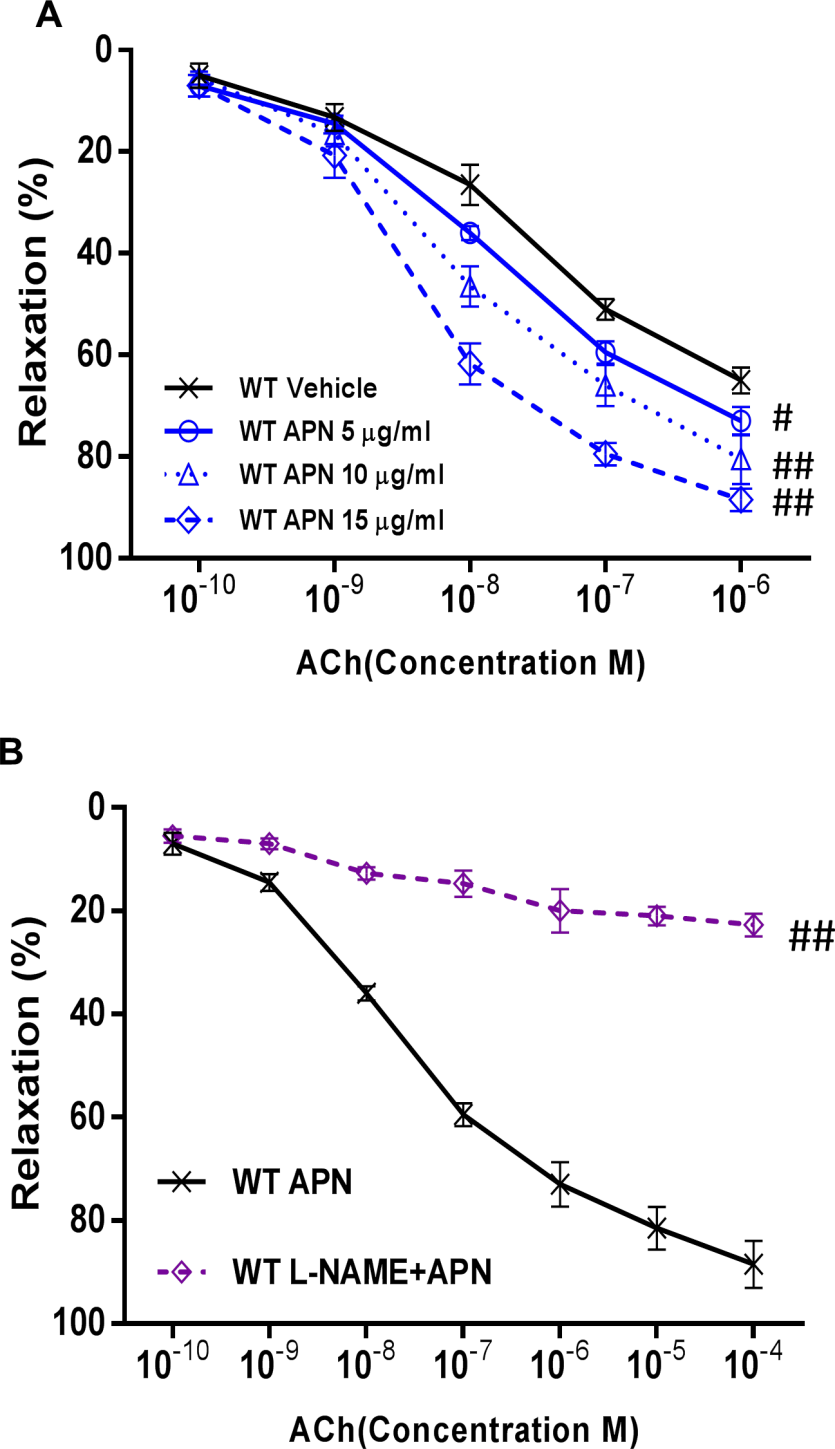
Physiologic APN doses facilitate ACh-induced vasorelaxation in an endothelium-dependent and NO-mediated manner. (A) Different physiologic APN levels (5, 10, 15μg/ml) significantly enhanced ACh-induced vasorelaxation in a concentration-dependent fashion. (B) APN-induced aortic vascular ring vasorelaxation is blocked after pre-treatment with L-NAME (NO synthesis inhibitor, 100 μmol/L). n = 5–7 mice/group. #,*P*<0.05; ##, *P*<0.01.

Aortic rings were isolated from wild type mice. Administration of APN at physiologic concentrations (up to 15μg/ml) resulted in no apparent vasodilatory effects (data not show). However, pretreatment of wild type mouse aortae with different physiologic APN levels (5, 10, 15μg/ml) significantly enhanced ACh (an established, endothelium-dependent vasodilatory agent)-induced vasorelaxation in dose-dependent fashion (5μg/ml APN 79.52±1.95% vasorelaxation vs control 51.91±2.02%, P<0.01, Fig 1A).

Vasodilation occurs physiologically by two mechanisms, namely enhanced endothelial sensitivity or enhanced smooth muscle responsiveness. To determine the manner by which APN enhances the vasodilatory effects of ACh upon endothelial cells, we employed L-NAME (100μmol/L, a NO synthase inhibitor). As seen in Fig 1B, pre-treatment with L-NAME abrogated APN-enhanced ACh-induced vasorelaxation (5 μg/ml APN treatment 87.63±2.24% vs L-NAME pretreatment then APN 20.57±1.66%, P<0.01). Together, these data suggest physiological-dose APN enhances the endothelial cell sensitivity in a NO-dependent manner.

### Knockout of adiponectin receptor 1 (AdipoR1), but not receptor 2 (AdipoR2), abolishes APN-enhanced ACh-induced vasorelaxation

**Fig 2:**
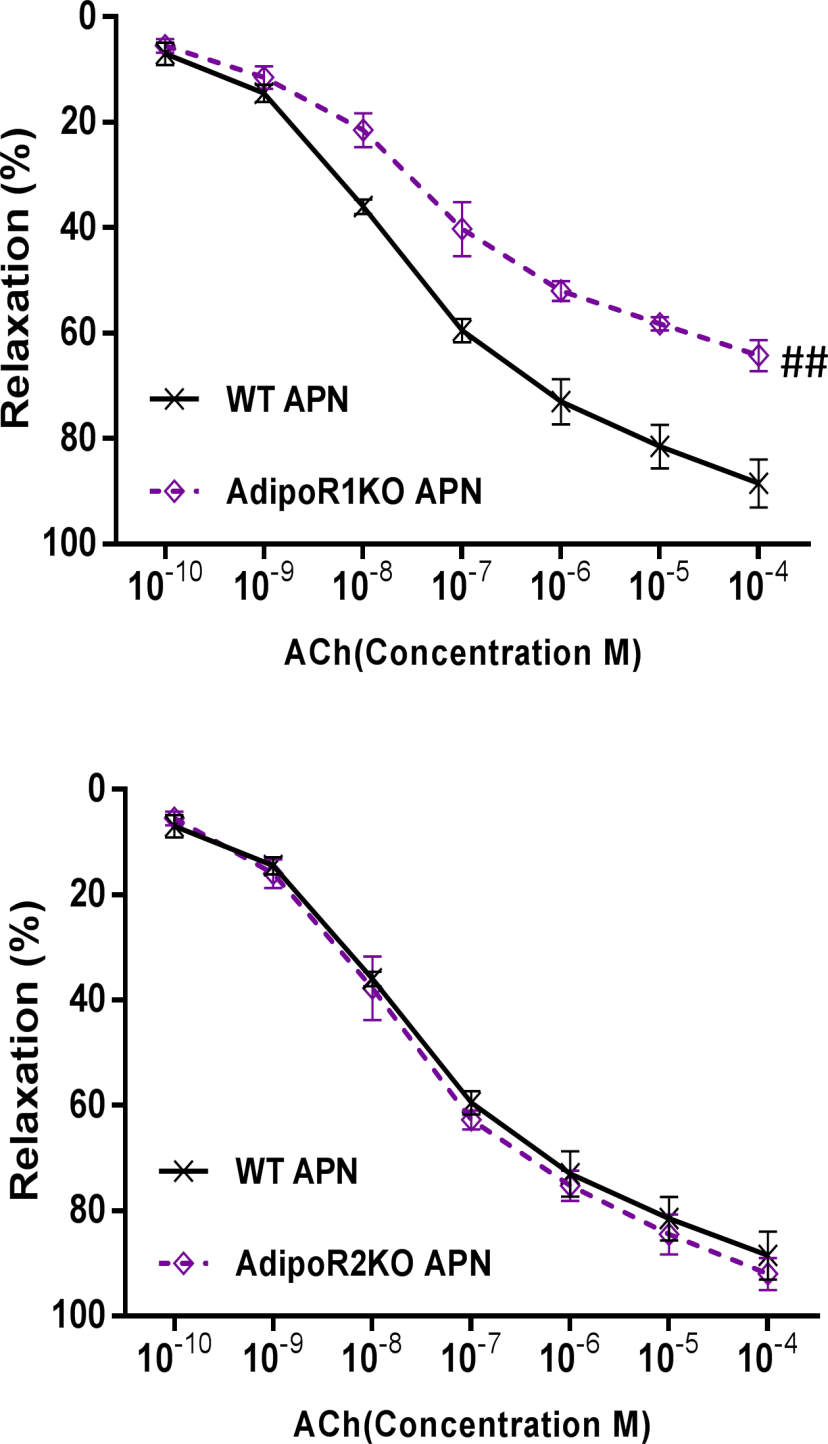
Knockout of adiponectin receptor 1 (AdipoR1), but not receptor 2 (AdipoR2), abolishes APN-enhanced ACh-induced vasorelaxation. (A) Enhanced vasorelaxation to ACh by APN (5μg/ml) was significantly decreased in AdipoR1-KO mouse aorta. (B) Enhanced vasorelaxation to ACh by APN (5μg/ml) was not affected in AdipoR2-KO mouse aorta. n = 5–7 mice/group. #, *P*<0.05; ##, *P*<0.01.

Having demonstrated physiologic doses of APN enhanced ACh-induced vasorelaxation in an NO-dependent manner, we next determined the adiponectin receptor subtype (AdipoR1 or AdipoR2) responsible. Administration of APN (5 μg/ml) in AdipoR1-KO mouse aorta significantly decreased ACh-induced vasorelaxation (WT 82.96±1.77% vs AdipoR1KO 58.37±2.01%, P<0.01, Fig 2A). However, APN-enhanced ACh-induced vasorelaxation remained intact in AdipoR2-KO aortae (Fig 2B). Together, these data support the involvement of AdipoR1 in APN-enhanced ACh-mediated vasorelaxation.

### Physiologic APN levels induced eNOS phosphorylation in an AdipoR1-dependent fashion

Having demonstrated physiologic doses of APN enhanced ACh-induced vasorelaxation via AdipoR1, we next investigated the responsible underlying molecular mechanisms. Acetylcholine activates endothelial nitric oxide synthase (eNOS), which increases NO release. In the next experiment series, we determined whether physiologic APN doses augment eNOS activation, thereby facilitating increased ACh-induced vasorelaxation.

**Fig 3:**
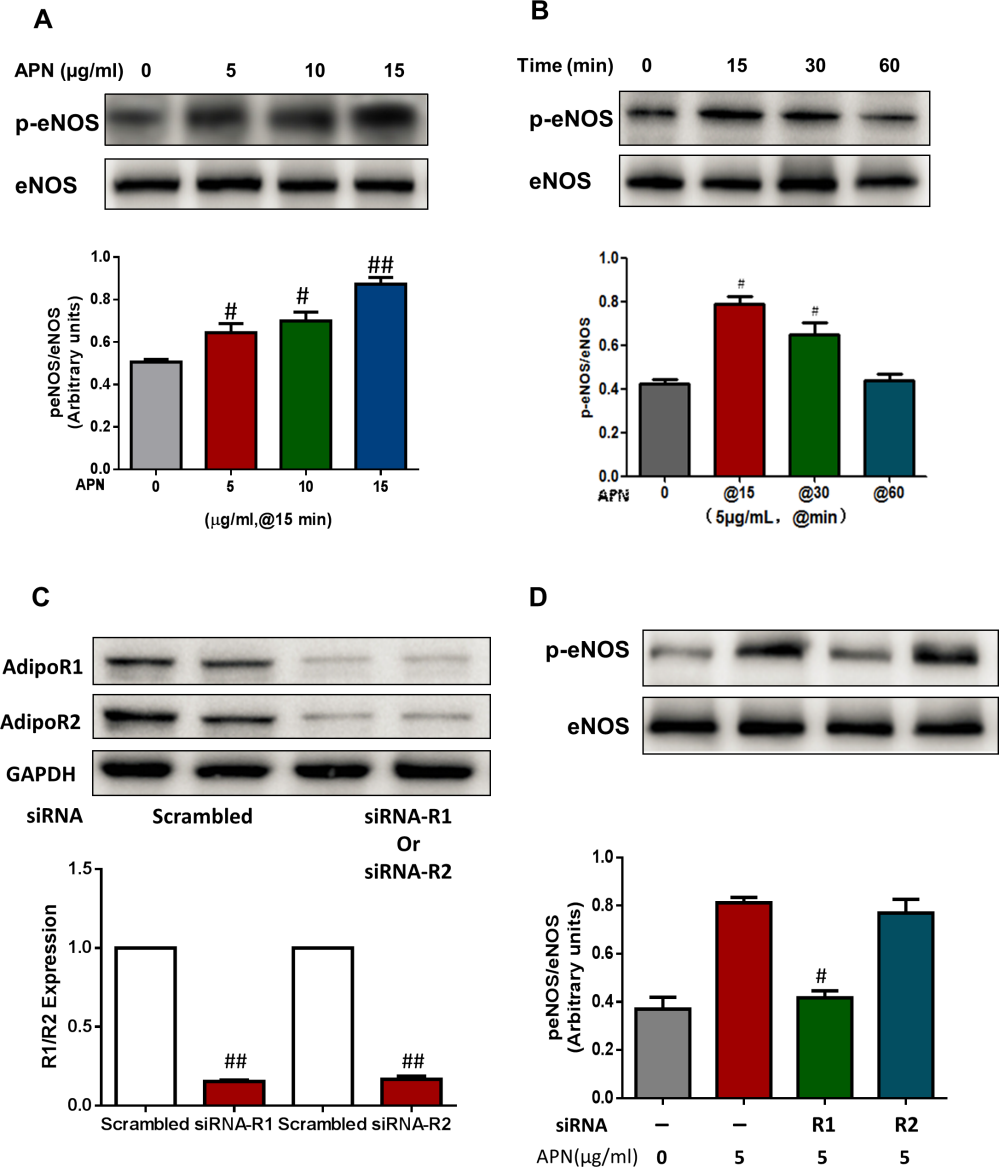
Physiologic APN levels induced eNOS phosphorylation in an AdipoR1-dependent fashion. HUVECs were incubated with different doses of APN (5, 10, and 15μg/ml) (A) and eNOS phosphorylation (Ser1177) was determined at different time periods (0, 15, 30, and 60 min) (B). (C) Western blot analysis confirmed successful knockdown of AdipoR1 and AdipoR2 by siRNA. (D) AdipoR1, but not AdipoR2, knockdown significantly attenuated APN-induced eNOS phosphorylation. n = 6–8 dishes/group. #, *P*<0.05; ##, *P*<0.01 vs respective control. R1, R2 represent AdipoR1 and AdipoR2, respectively.

HUVECs were incubated with different physiologic doses of APN (5, 10, and 15 μg/ml) for different time periods (0, 15, 30, and 60 minutes). eNOS phosphorylation at Ser^1177^ was determined. After a fixed 15-minute period of exposure, physiologic doses of APN increased eNOS phosphorylation in a dose dependent manner (Fig 3A). At a fixed 5 μg/ml concentration, APN-induced eNOS phosphorylation peaked after 15 minutes of exposure (Fig 3B).

To further determine the molecular mechanisms underlying physiologic dose APN-induced eNOS phosphorylation, siRNA was employed to downregulate endogenous AdipoR1 and AdipoR2 production. Western blot analysis confirmed successful suppression of endogenous AdipoR1 and AdipoR2 expression (72 hours siRNA transfection decreased AdipoR1 or AdipoR2 expression by 70–80%, Fig 3C). In Fig 3D, we redemonstrate APN (physiologic dose 5 μg/ml) increased eNOS phosphorylation (Lane 2), compared to control (Lane 1), and downregulation of AdipoR1 (Lane 3), but not AdipoR2 (Lane 4), blocked APN-induced eNOS phosphorylation. Taken together, these results support APN activates eNOS via AdipoR1 signaling.

### Cav-1 is required for AdipoR1-dependent APN-enhanced vasorelaxation

Caveolae are small (100 nm diameter) flask-like invaginations in the plasma membrane, providing spatial and temporal regulation of cell signaling events [14]. The caveolins, scaffolding proteins within the caveolae, compartmentalize and concentrate signaling molecules, and are responsible for endothelial function regulation [15, 16]. We have recently demonstrated the existence of a membrane-situated APN signalsome, containing APN receptor 1 (AdipoR1) and caveolin-1 (Cav-1), that facilitates APN signaling transduction [17]. Our study demonstrated direct interaction between AdiopR1 and Cav-1 (the primary caveolin subtype in HUVECs) [17].

The results presented in Figs 2 and 3 collectively suggest the pivotal role of AdipoR1 in APN-enhanced ACh-induced vasorelaxation. We next investigate whether the enhancement of ACh-induced vasorelaxation by physiologic concentrations of APN are dependent upon AdipoR1 association with Cav1.

**Fig 4:**
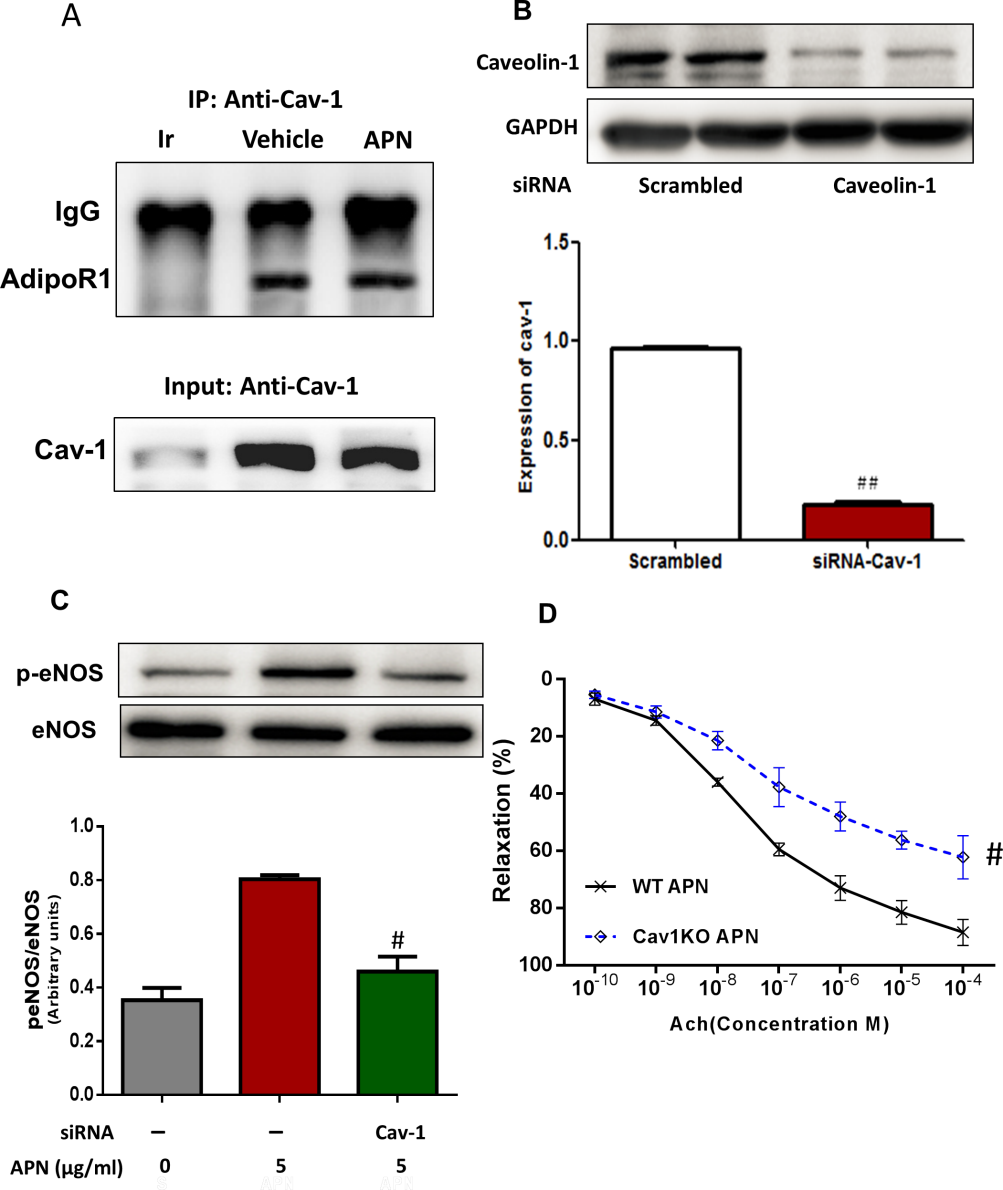
Cav-1 is required for AdipoR1-dependent APN-enhanced vasorelaxation. (A) Immunoblot demonstrating interaction of Cav1 with AdipoR1 in the presence or absence of APN (5μg/ml). (B) Western blot analysis confirmed successful knockdown of Cav-1 by siRNA. n = 6–8 dishes/group. (C) Knockdown of Cav-1 significantly attenuated APN-induced eNOS phosphorylation. (D) Cav-1 KO mouse aorta exhibited significantly decreased APN enhancement to ACh-induced vasorelaxation. Ir, irrelevant antibody. n = 5–7 mice/group. #, P<0.05; ##, P<0.01 vs respective control.

After administration of APN to HUVECs, an anti-Cav-1 antibody was employed to immunoprecipitate Cav-1 with its binding partner. The immunoblot in Fig 4A demonstrates AdipoR1 interacts with Cav-1, as previously demonstrated [17, 18]. Western blot analysis confirmed successful suppression of endogenous Cav-1 in HUVECs (72 hours siRNA transfection decreased Cav-1 by 89%, Fig 4B). In Fig 4C, we redemonstrate APN (physiologic dose 5 μg/ml) increased eNOS phosphorylation (Lane 2), compared to control (Lane 1), and downregulation of Cav-1 (Lane 3), blocked APN-induced eNOS phosphorylation. Finally, APN (5μg/ml)-enhanced ACh-induced vasorelaxation was partially blocked in Cav-1 KO mice (WT 83.39±2.19% vs Cav-1KO 20.57±1.66%, P<0.01, Fig 4D). Taken together, these results demonstrate eNOS activation by APN is dependent upon an intact AdipoR1/Cav-1 signalsome. Furthermore, this signalsome is necessary for APN-enhanced ACh-mediated vasorelaxation.

### Physiologic APN concentrations increased NO production in an AdipoR1 and Cav-1 dependent manner

**Fig 5:**
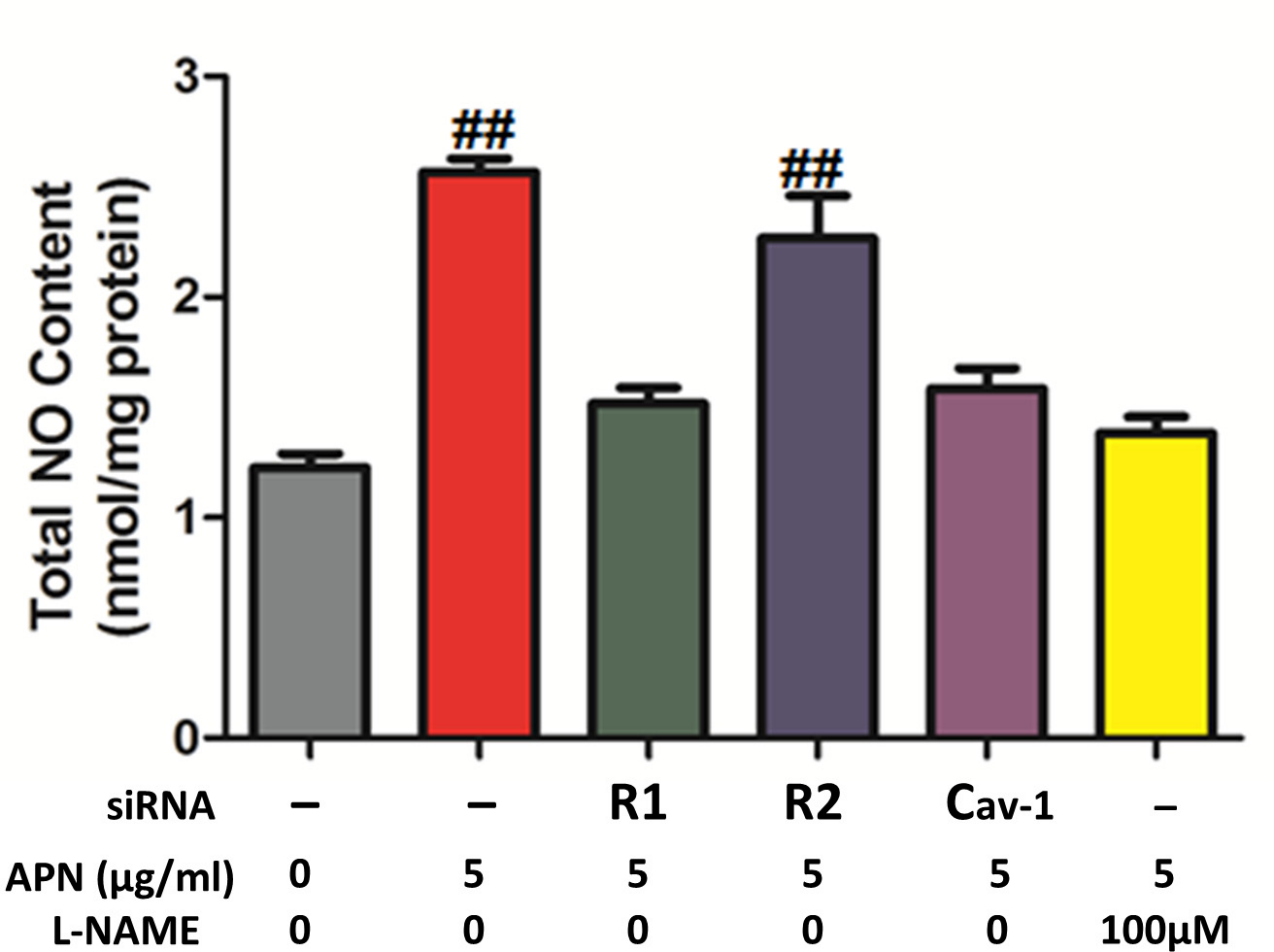
Physiologic APN concentrations increased NO production in an AdipoR1 and Cav-1 dependent manner. NO production in HUVEC medium was determined after incubation with vehicle or APN (5μg/ml) for 15 minutes. Knockdown of AdipoR1 (but not AdipoR2), knockdown of Cav-1, and treatment with L-NAME (100 μmol/L) abolished APN-induced NO production enhancement. n = 6–8 dishes/group. ##, *P*<0.01 vs respective control. R1, R2 represent AdipoR1 and AdipoR2, respectively.

To further provide evidence supporting our study’s experimental data, we directly measured total NO content in cell medium after a physiologic dose of APN (5μg/ml) was administered to various HUVEC cell types for 15 minutes (Fig 5). APN (Lane 2) increased total NO content in cell medium compared to control (Lane 1, APN 2.57±0.06 vs 1.3±0.10 control, P<0.01). APN did not significantly increase total NO content in AdipoR1 KO cell medium (Lane 3, 1.56±0.06), but did so in AdipoR2 KO cell medium (Lane 4, 2.41±0.17). APN did not significantly increase total NO content in Cav-1 KO cell medium (Lane 5, 1.34±0.12). Finally, when L-NAME (dose 100 μM) was administrated concomitantly with APN to WT cells, total NO in the cellular medium remained unchanged (Lane 6, 1.12±0.17). Taken together, our data supports the AdipoR1/Cav-1 signaling pathway is essential in APN (at a physiologically relevant dose)-mediated NO production.

## Discussion

**Fig 6:**
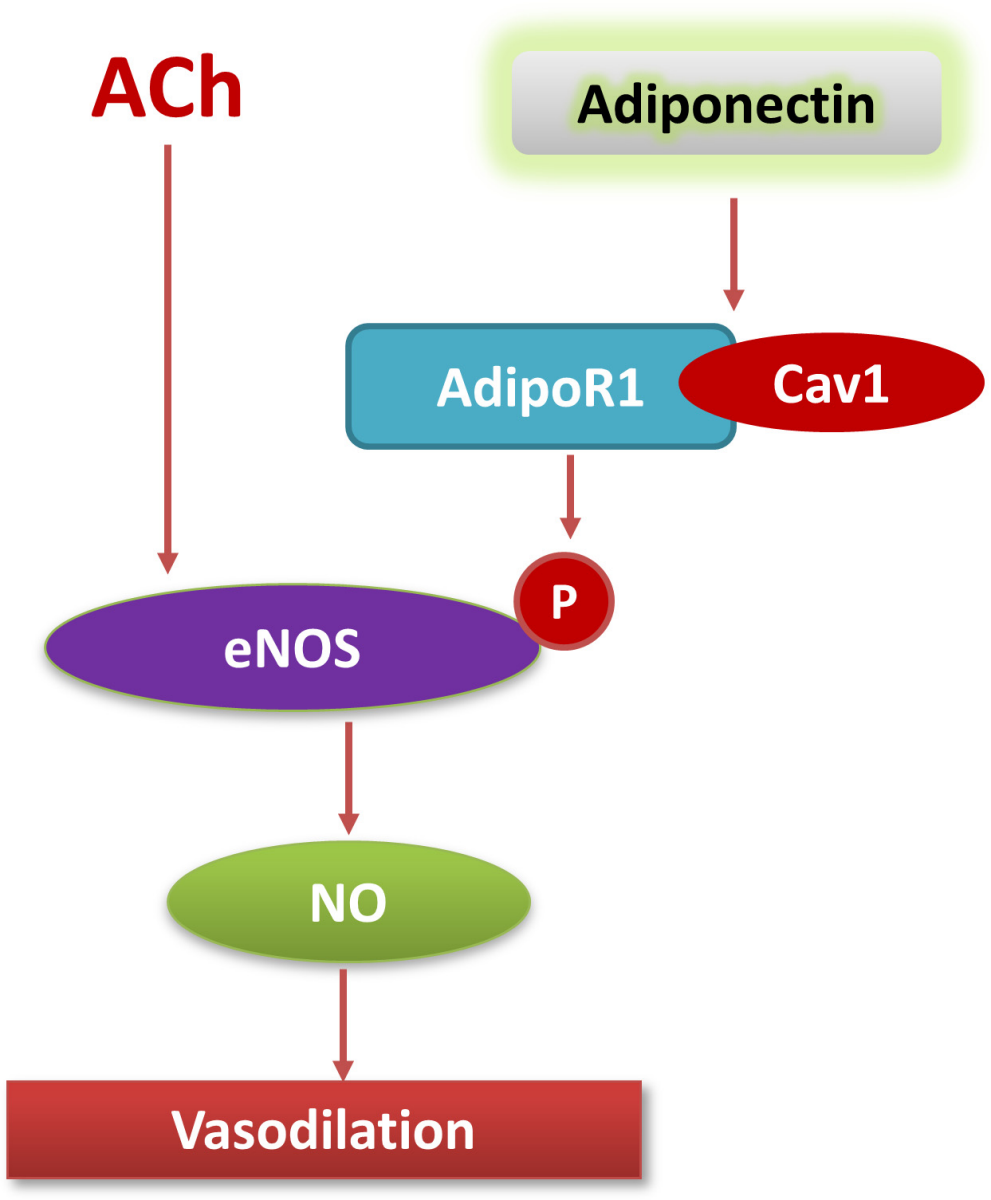
Schematic of the mechanisms underlying APN-induced vasodilation.

This study investigated the role of physiological adiponectin concentrations in endothelial dysfunction associated with hypertension. We demonstrated for the first time physiological adiponectin concentrations facilitated endothelium-dependent vasorelaxation and endothelial NO production by phosphorylating eNOS in an AdipoR1 and Cav-1 dependent manner (Fig 6).

Of primarily adipocyte origin, circulating adiponectin accounts for nearly 0.01% of all plasma proteins [19]. Physiological APN concentration is approximately 5–10 μg/ml [20, 21]. The basic structure of full-length form of adiponectin (fAPN) consists of four domains: an N-terminal signal sequence, a short region with no homology to any known protein, a collagen-like domain, and a C-terminal globular domain. The human and murine globular domain of adiponectin (gAPN) shares 91% conservation, indicating this domain preserves a vital biological function [22]. Acting as a protein hormone, full length APN (fAPN) modulates numerous metabolic processes [23], sensitizes insulin sensitivity [17, 24] as well as protects against atherosclerosis [25]. Clinical studies have demonstrated an evident association between adiponectin and hypertension [7, 8, 26], and supra-pharmacological doses of fAPN cause direct vasodilation [11, 12]. Most research efforts have centered upon gAPN, because of its important biological function. However, in the present study, we show here for the first time that although physiologic fAPN levels (5μg/ml) are insufficient to be vasodilator to directly dilate vessel itself, it enhances vasorelaxative response to vasodilatory factors, such as ACh. To minimize known gender discrepancies in vascular vasodilatory response [27] (particularly to Ach [28]), only male mice were employed in the current study.

Previously, we have demonstrated the APN signalsome, containing AdipoR1 and Cav-1, facilitates APN signaling transduction. In the current study, AdipoR1 knockout mice exhibit reduced fAPN-mediated ACh-induced vasorelaxation in aortic rings. However, in AdipoR2 knockout mice, there was no difference in fAPN-promoted ACh-induced vasorelaxation in aortic rings compared to control. These results emphasize AdipoR1, not AdipoR2, is critically involved in the physiologically relevant adiponectin concentration-mediated vasorelaxation.

The endothelium abundantly expresses Cav-1, a protein responsible for transcellular transport in endothelial cells, regulation of endothelial permeability, and acceleration of atherosclerosis [29–32]. Moreover, caveolin-1 is also responsible for signal transduction via its scaffolding domain. Our current study demonstrates that both ACh-mediated vasodilation and NO production were inhibited in aortic rings isolated from Cav-1 knockout mice. siRNA-mediated suppression of Cav-1 expression significantly attenuated APN-induced eNOS phosphorylation and NO production. Furthermore, mice overexpressing adiponectin exhibit increased Cav-1 levels in adipocytes [32]. Recently, it was reported high glucose/high lipids impair the effect of adiponectin upon vascular function via inhibition of the AdipoR1/Cav-1 signalsome [17]. Taken together, these data show that AdipoR1/Cav-1 complex is essential for APN signaling transduction.

Nitric oxide (NO) is pivotal in the regulation of endothelial function. Released from the endothelium itself, NO is synthesized from L-arginine by endothelial NO synthase (eNOS). Phosphorylation of eNOS leads to its activation, and NO production [33]. In adiponectin knockout vessels, eNOS expression is unchanged, but NO production and eNOS phosphorylation significantly decreased [9]. The present study provides the first evidence that physiologically relevant APN levels promoted vasorelaxative response to ACh in WT aortic rings. L-NAME administration inhibited APN-mediated ACh-induced aortic ring vasorelaxation. Our experiments involving HUVECs confirmed physiologic APN levels enhance eNOS phosphorylation and NO production in vitro. Together, these results support physiological full-length APN concentrations can cause endothelium-dependent and NO-mediated vasorelaxation.

Endothelial dysfunction is one of the causes of hypertension, and the association between hypoadiponectinemia and hypertension is well documented [26, 33, 34]. In the current study, we elucidated the mechanism responsible for physiologically relevant concentrations of APN levels-induced vasorelaxation, by inducing NO production via AdipoR1/Cav-1 signaling. Pathologic conditions impairing proper APN signaling, such as diabetes and metabolic syndrome, may therefore initiate hypertension development. Therapies restoring the APN signaling axis may represent novel modalities preventing hypertension onset and its costly complications.
